# Simple 3,4-Dihydroxy-*L*-Phenylalanine Surface Modification Enhances Titanium Implant Osseointegration in Ovariectomized Rats

**DOI:** 10.1038/s41598-017-18173-5

**Published:** 2017-12-19

**Authors:** Ting Ma, Xi-Yuan Ge, Ke-Yi Hao, Bi-Ru Zhang, Xi Jiang, Ye Lin, Yu Zhang

**Affiliations:** 1 Department of Oral Implantology, Peking University School and Hospital of Stomatology, Beijing, 100081 People’s Republic of China; 2 Central Laboratory, Peking University School and Hospital of Stomatology, Beijing, 100081 People’s Republic of China

## Abstract

Osteoporosis presents a challenge to the long-term success of osseointegration of endosseous implants. The bio-inspired 3,4-dihydroxy-*L*-phenylalanine (Dopa) coating is widely used as a basic layer to bind osteogenetic molecules that may improve osseointegration. To date, little attention has focused on application of Dopa alone or binding inhibitors of bone resorption in osteoporosis. Local use of a bisphosphonate such as zoledronic acid (ZA), an inhibitor of osteoclast-mediated bone resorption, has been proven to improve implant osseointegration. In this study, ovariectomized rats were divided into four groups and implanted with implants with different surface modifications: sandblasted and acid-etched (SLA), SLA modified with Dopa (SLA-Dopa), SLA modified with ZA (SLA-ZA), and SLA modified with Dopa and ZA (SLA-Dopa + ZA). Measurement of removal torque, micro-computed tomography and histology revealed a greater extent of bone formation around the three surface-modified implants than SLA-controls. No synergistic effect was observed for combined Dopa + ZA coating. Microarray analysis showed the Dopa coating inhibited expression of genes associated with osteoclast differentiation, similarly to the mechanism of action of ZA. Simple Dopa modification resulted in a similar improvement in osseointegration compared to ZA. Thus, our data suggest simple Dopa coating is promising strategy to promote osseointegration of implants in patients with osteoporosis.

## Introduction

Endosseous implants are widely applied for direct orthodontic and orthopedic anchorage, and their success mainly depends on the stability of osseointegration^1^. Osseointegration, defined as the stable anchorage of an implant achieved by direct bone-to-implant contact^2^, is a critical determinant of internal fixation and functional loading, both of which are influenced by the density and quality of the surrounding bone. Clinically, it is difficult to achieve optimal osseointegration in “soft bone” with a low mineralization density^1,3^, such as in patients with osteoporosis. According to the World Health Organization, 300 million people worldwide are affected by osteoporosis^4^, which is characterized by reduced bone mass, decreased mechanical strength and an increased risk of fracture. Patients with osteoporosis often have a lower jaw bone density due to an imbalance between osteoblasts and osteoclasts^5^. The endosseous implant loosening rate can be as high as 25% in osteoporotic bone^6,7^; therefore, osteoporosis poses a challenge to the long-term success of osseointegration for stable internal fixation.

Surface properties are key factors that affect the osseointegration of implants, and various surface modifications have been explored to improve osseointegration, especially under compromised bone-healing conditions^8^. Previous studies evaluated the merits of different implant surface coatings in animal models of osteoporosis, including calcium phosphate, collagen type-I, strontium-substituted hydroxyapatite coatings and other surface coatings^9–11^. However, it is still necessary to improve the long-term stability of implants in the clinically compromised scenario of osteoporosis.

The adhesive ability of mussels under aqueous conditions mainly depends on *Mytilus edulis* foot protein 5 (Mefp-5), which contains consecutively repeated 3,4-dihydroxy-*L*-phenylalanine (Dopa) motifs^12,13^. Mussel-derived Dopa coatings have been widely applied as a basic layer to functionally modify a variety of surfaces, such as binding antibodies, peptides, enzymes, growth factors or nano-particles, to modulate their biological effects^14–18^. Generally, Dopa coating has been applied in combination with factors that promote osteogenesis for implant osseointegration^19^. However, bone metabolism is influenced by the balance between osteogenesis and bone resorption; osteoporosis is characterized by increased osteoclast activity. The effect of Dopa coating alone or binding inhibitors of bone resorption on osseointegration under osteoporotic conditions are largely unknown.

**Figure 1:**
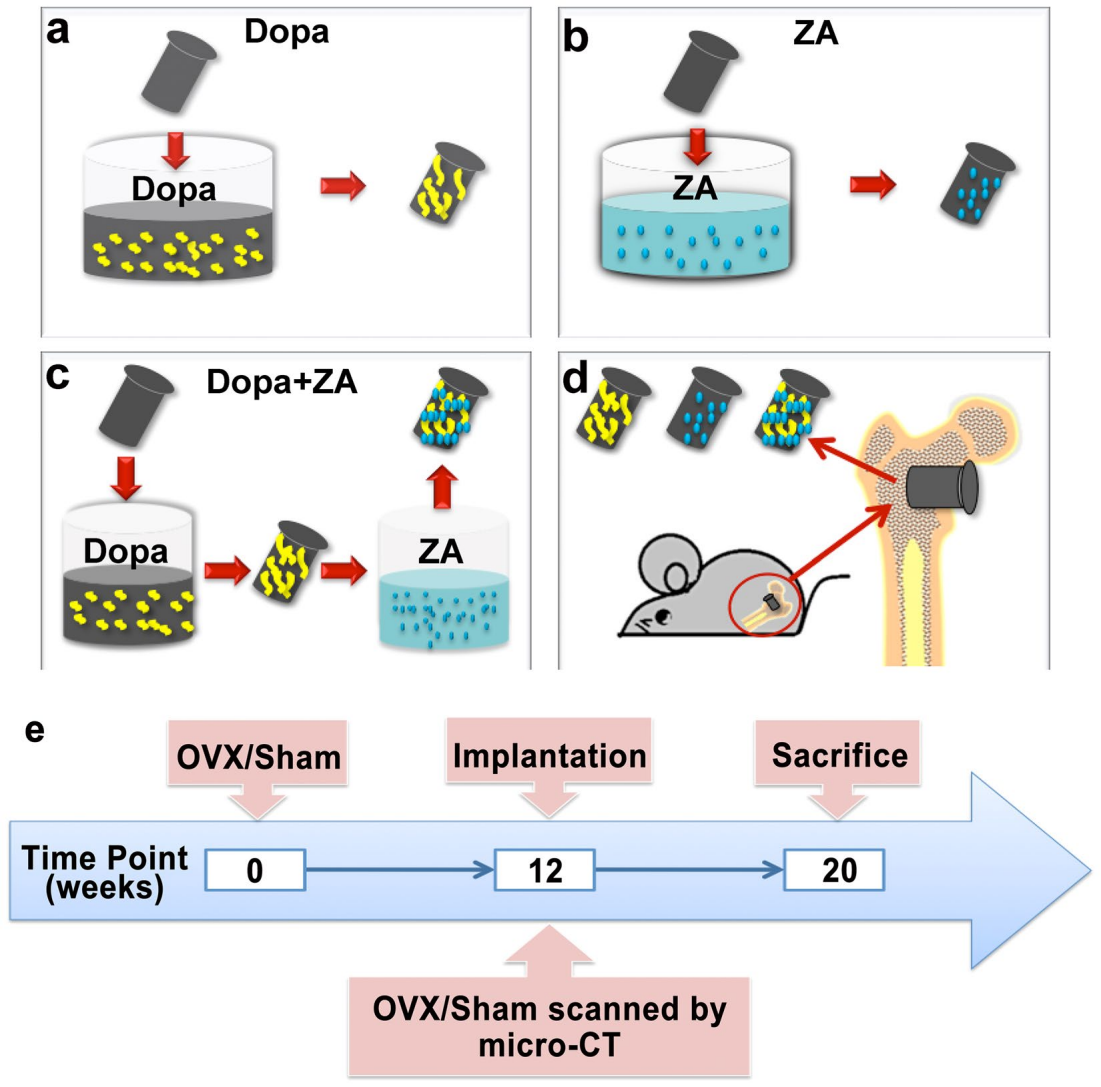
Schematic illustration of the surface modification (**a**–**c**) and implantation (**d**) of titanium implants coated with Dopa, ZA or Dopa combined with ZA. (**e**) Timeline of the animal surgery.

Bisphosphonates function as potent inhibitors of osteoclast-mediated bone resorption through several mechanisms, including inducing apoptosis of osteoclasts and inhibiting osteoclast maturation, differentiation and osteoclastic activity^20^. Local application of bisphosphonates has attracted attention as a strategy to enhance local treatment efficiency, reduce side effects and promote osseointegration of titanium implants in humans^21,22^. In this study, we aimed to determine whether application of Dopa coating alone or in combination with zoledronic acid (ZA), a third generation bisphosphonate, improves the osseointegration of titanium implants in a model of osteoporosis in ovariectomized rats (Fig. 1). We also explored the underlying mechanisms of action of the Dopa coating during the osseointegration of titanium implants using microarray analysis.

## Results and Discussion

Osteoporosis is an osteometabolic disease in which the balance between osteoblasts and osteoclasts is impaired^11,23^. Osteoporosis is characterized by reduced bone mass due to progressive bone resorption and micro-architectural changes, and presents a challenge to successful osseointegration. Biopolymer coatings represent a simple and promising approach to improve the osseointegration and stability of implant materials. Polymerized Dopa film has been used in biomedical research as an intermediate layer to immobilize other biofunctional molecules. Though Dopa coating enhanced osteogenesis when combined with peptides, growth factors or other functionalized bio-molecules as previously reviewed^24,25^, the influence of this biopolymer alone or in combination with osteoclast inhibitors on osseointegration under osteoporotic conditions *in vivo* remain to be elucidated.

**Figure 2:**
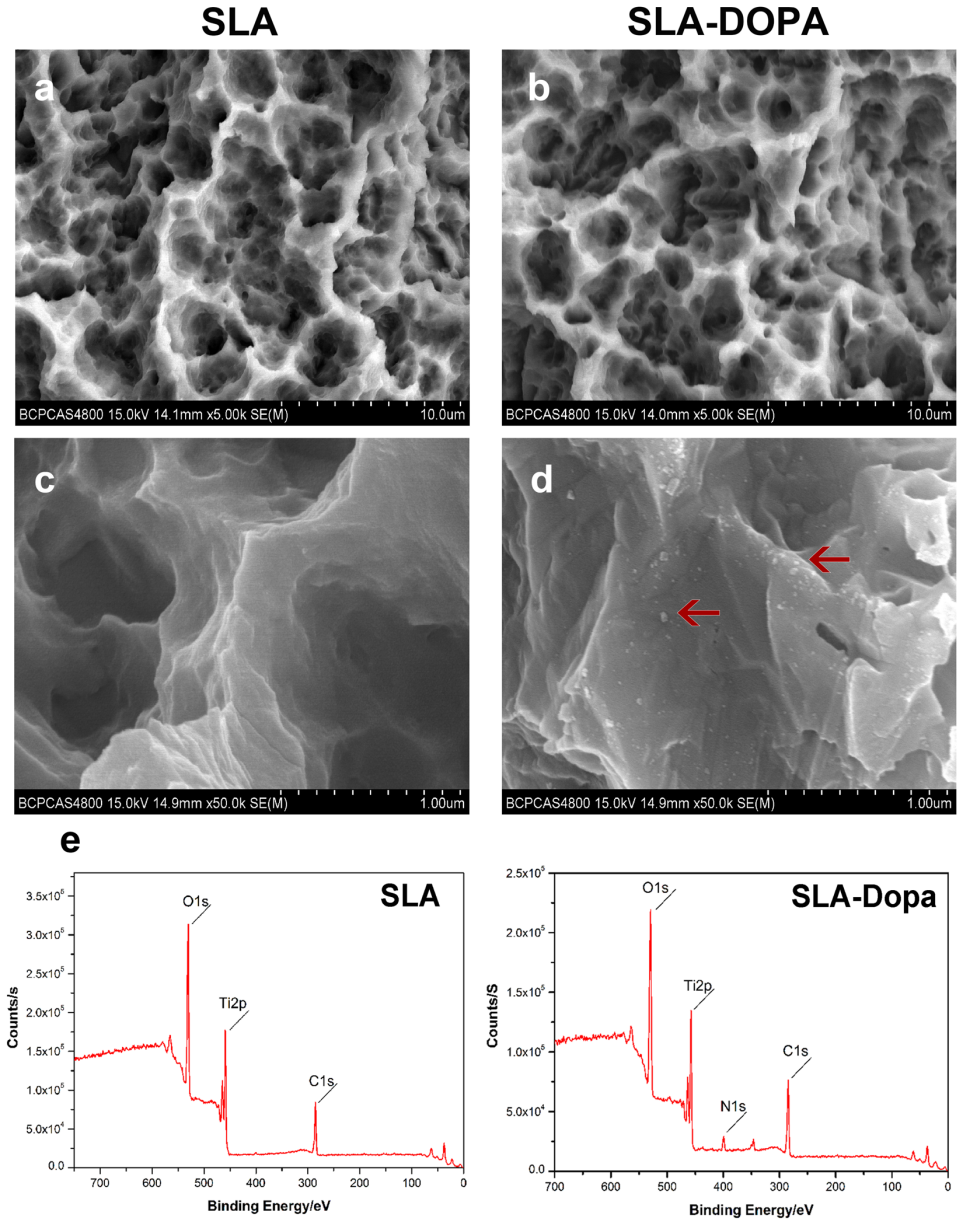
Characterization of the surface of the SLA and SLA-Dopa titanium implants. (**a**,**b**,**c**,**d**) SEM showed partial aggregates of polymerized Dopa (indicated by red arrows) were located between or in the cavities of the Dopa-modified SLA surfaces. (**e**) XPS analysis showed the Dopa-coated sample contained a peak corresponding to nitrogen (N1s), attributed to the amine group of Dopa.

Scanning electron microscopy (SEM) revealed that SLA resulted in a micro-roughened surface topography containing large cavities due to the alumina grit-blasting and acid-etching (Fig. 2a,b). Partial aggregates of polymerized Dopa were observed between or in the cavities of the Dopa-modified SLA surface on SEM (Fig. 2c,d). Dopamine particles 10–100 nm wide to 10–25 nm high have previously been observed on a polished surface^26,27^. Assessment of surface atomic composition using X-ray photoelectron spectroscopy (XPS) revealed that the Dopa coating clearly increased the nitrogen peak intensity (N1s) at 400 eV, corresponding to the amine group of Dopa (Fig. 2e). The changes in the percentages of functional elements demonstrated the titanium discs were successfully coated with Dopa.

**Figure 3:**
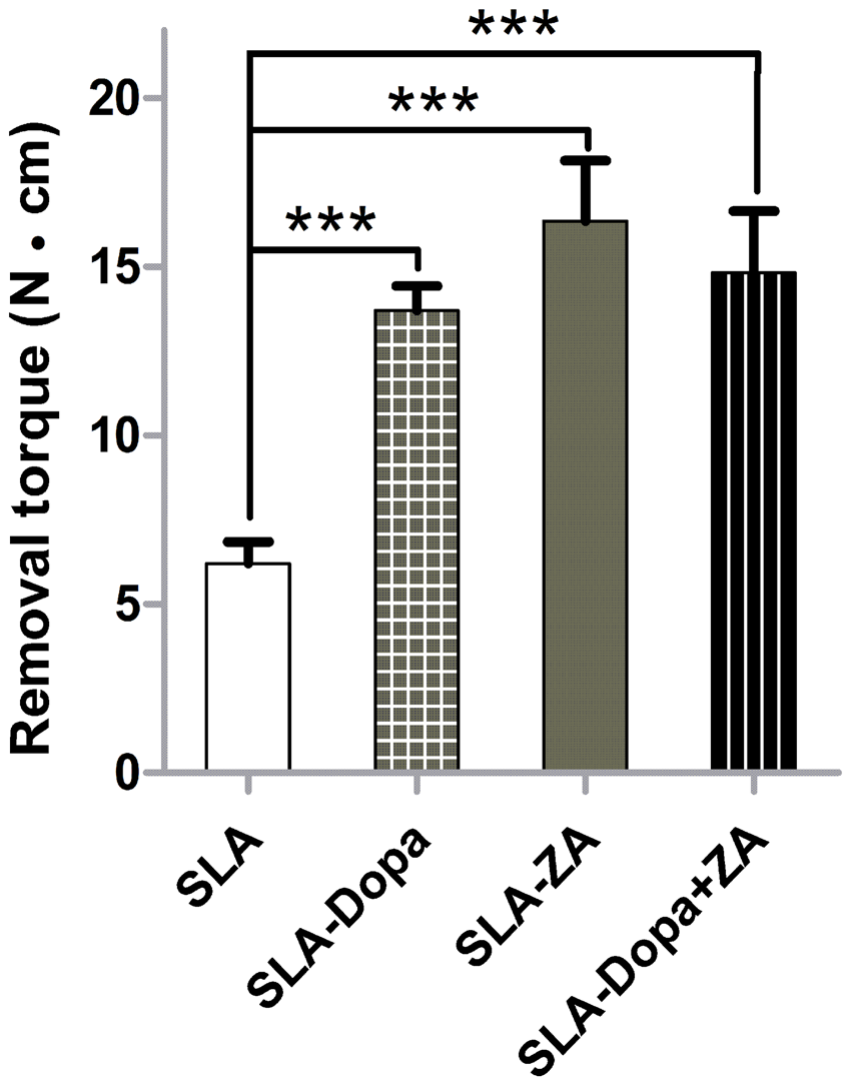
Removal torque for SLA titanium implants with different coatings at 8 weeks after implantation. N**·**cm, Newton centimeters (***P < 0.001, *n* = 5).

The peak removal torque value measured in Newton centimeters (N**·**cm) reflects the shear strength of the interface between an implant and the surrounding bone tissues. In this study, SLA titanium implants modified with Dopa, ZA or Dopa combined with ZA were implanted into rats in which osteoporosis was induced by OVX (Fig. S1). The three surface-coated implants (SLA-Dopa, SLA-ZA, SLA-Dopa + ZA) had significantly higher removal torque values than control SLA implants at 8 weeks after implantation (*P* < 0.001; Fig. 3), with no significant difference between the SLA-Dopa, SLA-ZA and SLA-Dopa + ZA implants. These results indicate that the Dopa coating markedly improved mechanical fixation of the implant to the surrounding bony structure.

**Figure 4:**
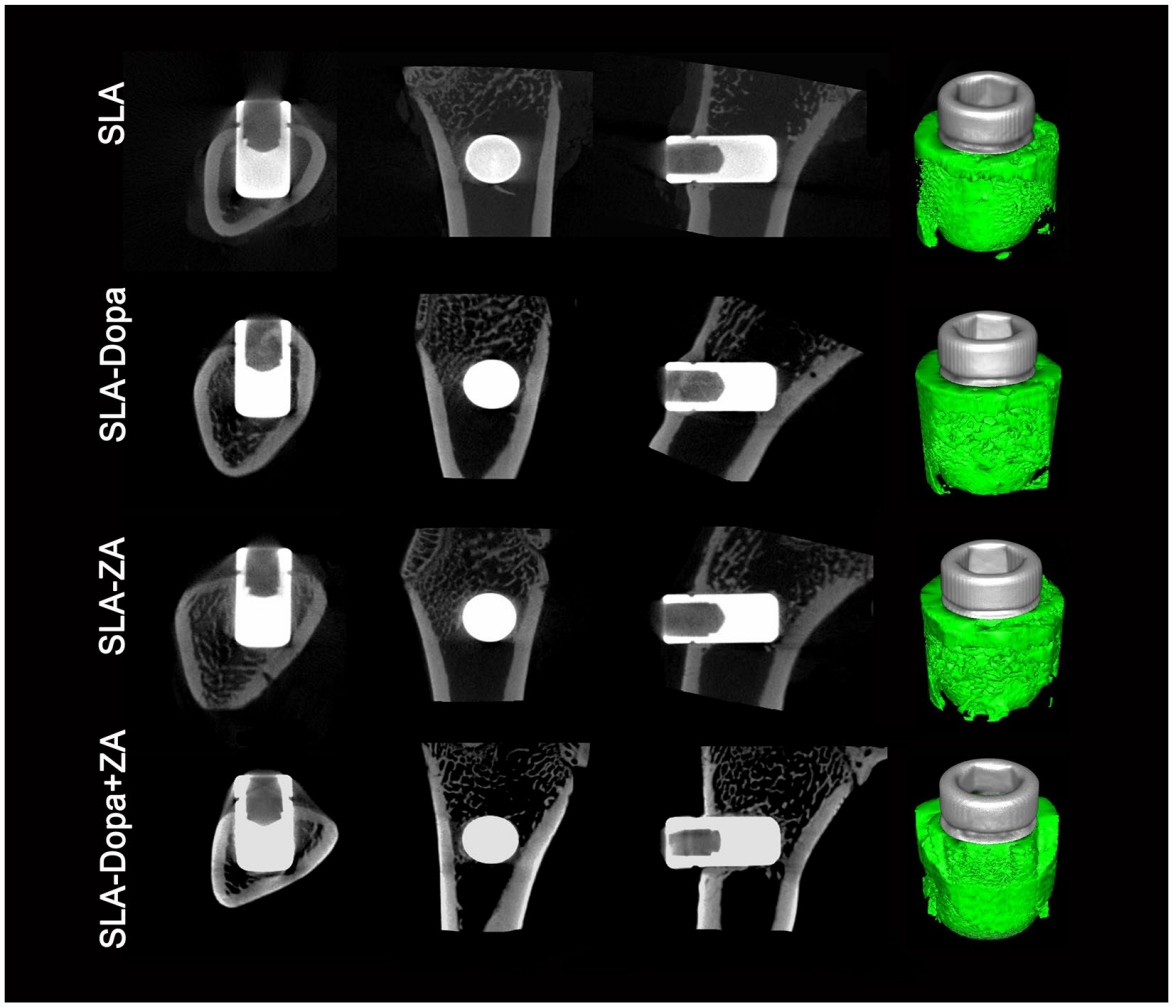
Typical micro-CT images (2D slices) of bone formation and 3D micro-CT reconstructions of trabecular bone formation around implant sites; trabecular bone structure can be visualized on the implant surface.

**Figure 5:**
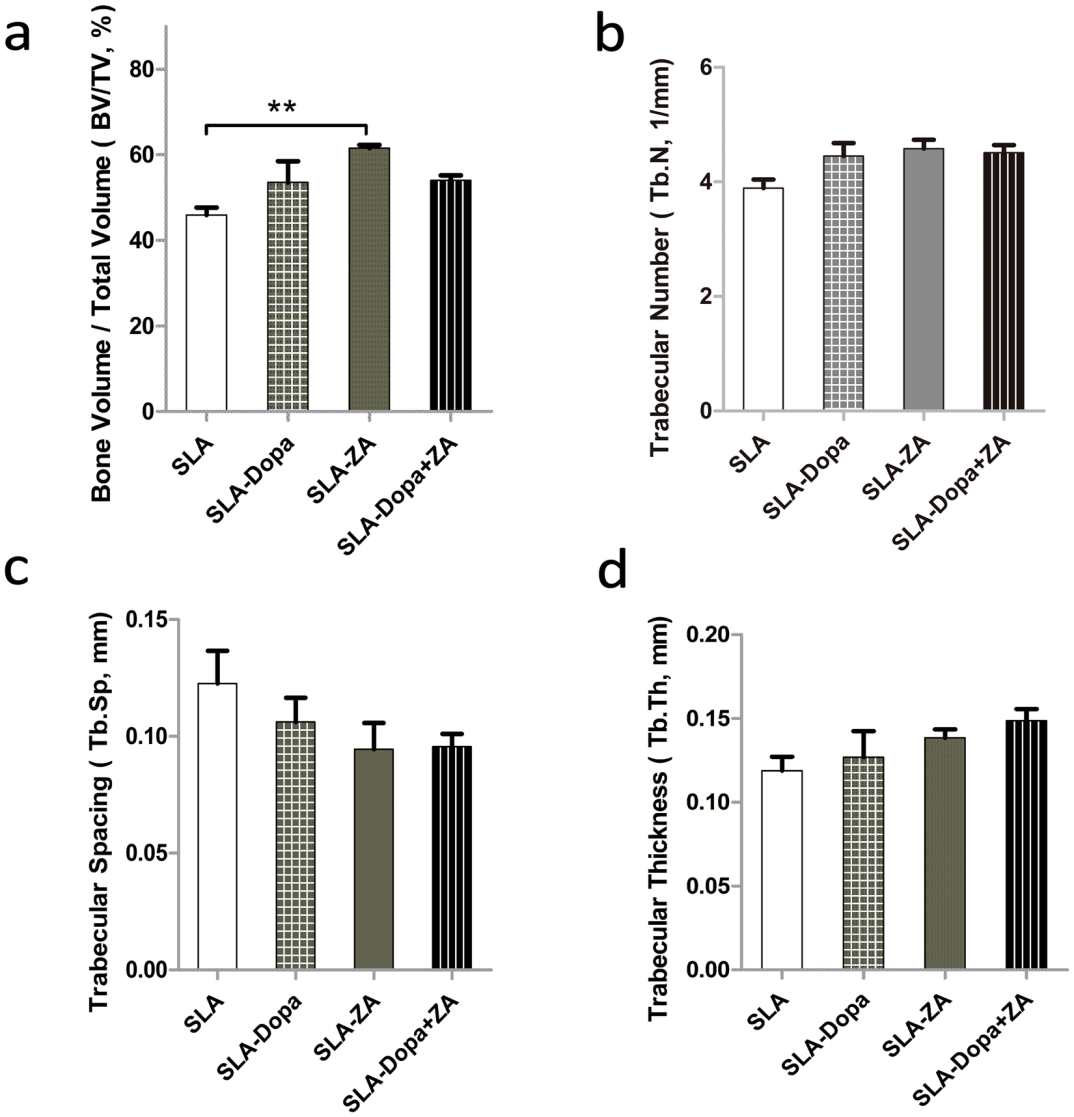
Quantitative comparison of bone micro-architecture parameters by micro-CT. The micro-CT images in the cylindrical volume of interest (VOI) were analyzed to calculate bone micro-architecture parameters. (**a**) Ratio of bone volume to total volume (BV/TV). (**b**) Trabecular numbers (Tb.N). (**c**) Trabecular spacing (Tb.Sp). (**d**) Trabecular thickness (Tb.Th). Error bars are mean ± SD; **P < 0.01.

Two-dimensional micro-CT analysis revealed a greater extent of bone formation around the SLA-Dopa, SLA-ZA and SLA-Dopa + ZA implants than the control SLA implants. Moreover, three-dimensional (3D) micro-CT reconstructions indicated increased trabecular bone formation occurred around the SLA-Dopa, SLA-ZA and SLA-Dopa + ZA implants compared to the SLA implants (Fig. 4). Micro-CT images enable quantitative analysis of bone: BV/TV is used to evaluate relative changes in bone volume density, and Tb.N, Tb.Sp and Tb.Th are key measures of microstructural bone configuration. BV/TV was significantly higher in the SLA-ZA group than the SLA group (*P* < 0.05; Fig. 5a). Tb.Sp was higher and Tb.Th and Tb.N were lower for the three groups of modified implants compared with the control SLA implants, although these differences were not statistically significant; this may related to the small sample size for these animal experiments (Fig. 5b,c,d).

**Figure 6:**
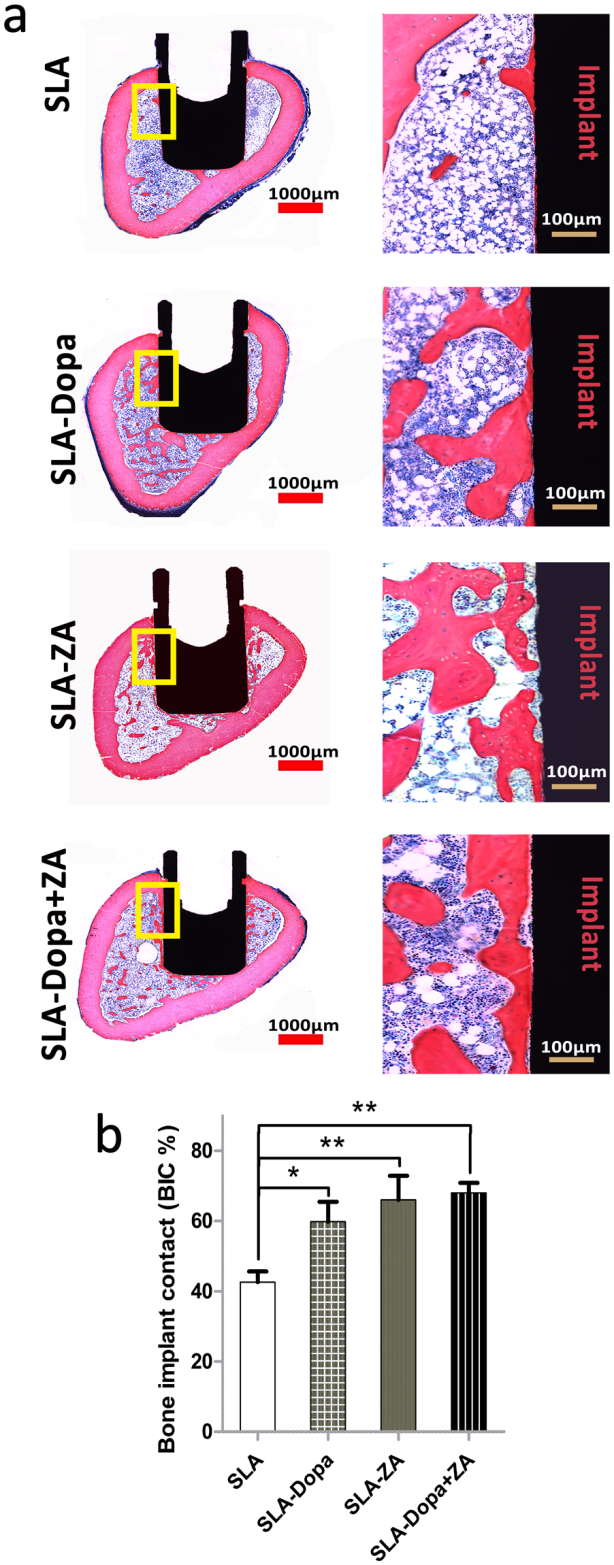
(**a**) Ground sections illustrating bone formation around the implants. Bars are 1000 μm (left) and 100 μm (right) in expanded images. (**b**) Histograms depicting bone to implant contact (BIC) percentage. Data are mean ± SD; *P < 0.05, **P < 0.01 vs. SLA implants.

Histological and histomorphometric analysis of ground sections confirmed bone remodeling had occurred around all implants at 8 weeks after implantation. Microscopically, the implants were predominantly in contact with cortical bone along the upper neck of the implant and in tight contact with trabecular bone (newly formed bone) around the body of the implant. New bone formation along the implant surface was enhanced in the SLA-Dopa, SLA-ZA and SLA-Dopa + ZA groups compared with the SLA group (Fig. 6a). Histological analysis and quantification of average BIC values indicated a higher degree of osseointegration occurred for the SLA-Dopa, SLA-ZA and SLA-Dopa + ZA implants than control SLA implants, with no significant difference among the three groups of surface-modified implants (*P* < 0.05; Fig. 6b).

The effects of bisphosphonates on osteoclasts have been well-described^20^. The first clinical study of humans receiving ZA-coated implants reported increased stability after 6 months of submerged healing^28^. Though Dopa modification is usually applied as a basic layer for further surface modifications, our histological and histomorphometric analyses showed that simple modification with Dopa film resulted in a similar enhancement of implant osseointegration in osteoporotic rats as coating with ZA. Moreover, no synergistic effect was observed between ZA and Dopa in the SLA-Dopa + ZA group compared with implants coated with Dopa alone or ZA alone.

**Figure 7:**
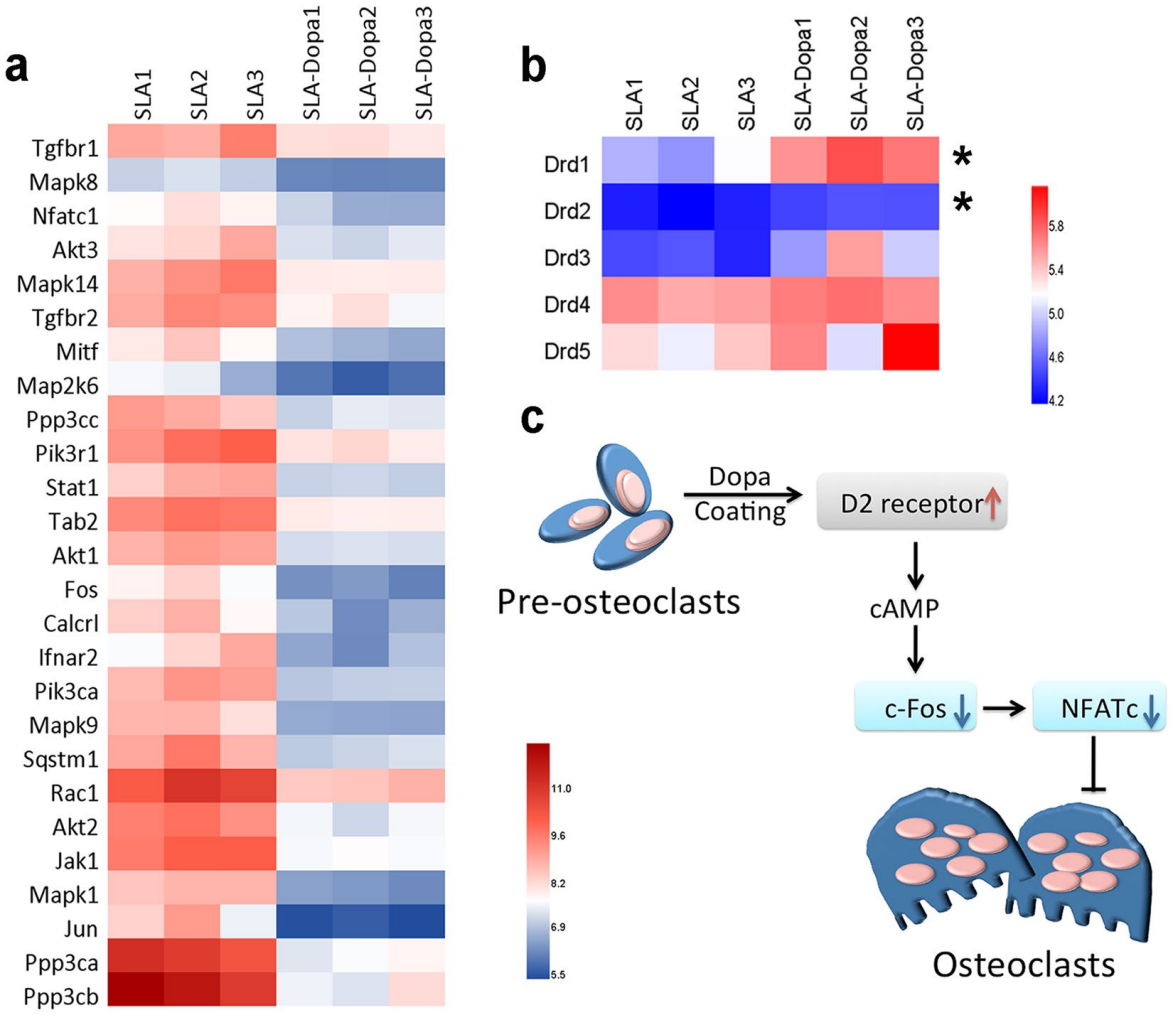
Microarray analysis of gene expression around implants with or without Dopa modification. (**a**) Heat maps for differential expressed genes associated with osteoclast differentiation. Differences were considered significant if the fold change was >2 or <0.5 and *P* < 0.05. Red represents high expression; blue, low expression. (**b**) Heat maps of the differential expression of the genes encoding five dopamine receptors (*P < 0.05). (**c**) Schematic illustration of the proposed mechanism by which Dopa modification influences genes related to osteoclast differentiation. Pre-osteoclasts are influenced by D2 receptors and the downstream genes.

To further explore the mechanisms underlying the effect of Dopa coating on enhanced osseointegration in osteoporotic rats, microarray analysis of RNA isolated from the bone around the implants in the femurs was performed to compare the gene expression patterns in the SLA and SLA-Dopa groups. Our analysis focused on genes associated with osteoblast and osteoclast activity. No significant differences were observed for genes related to osteoblast differentiation, including alkaline phosphatase (*Alpl*), bone morphogenetic protein 2 (*Bmp2*) and runt-related transcription factor 2 (*Runx2*; *P* > 0.05; data not shown). However, based on the Kyoto Encyclopedia of Genes and Genomes, 26 genes that participate in the osteoclast differentiation pathway (rno04380) were differentially expressed (fold change [fc] > 2 or < 0.5, *P* < 0.05; Fig. 7a). For example, nuclear factor of activated T-cells, cytoplasmic 1 (*NFATc1*) and c-Fos were downregulated in the SLA-Dopa group in comparison with the SLA group. *NFATc1*, a member of the nuclear factor of activated T cell family of transcription factors, is implicated in osteoclast differentiation^29^. A lack of c-Fos expression has been reported to result in blockade of osteoclast lineage differentiation^30,31^. Downregulation of *NFATc1* and *c-Fos* suggest osteoclast differentiation was inhibited in the Dopa-modified group. Dopamine is a major catecholamine neurotransmitter. The five dopamine receptors can be classified into two subfamilies: D1-like (D1R, D5R) and D2-like receptors (D2R, D3R and D4R) based on pharmacological modulation of cyclic adenosine monophosphate (cAMP)^32^. A previous report indicated D2R may inhibit *NFATc1* and *c-Fos* gene expression to suppress osteoclastogenesis^33^. In this study, the genes encoding both D1R and D2R were significantly upregulated in the SLA-Dopa group (*P* < 0.05; Fig. 7b). A previous study demonstrated that dopamine and dopamine D2-like receptor agonists, but not a D1-like receptor agonist, lower the intracellular cAMP concentration and suppress *c-Fos* and *NFATc1* gene expression in human osteoclast precursor cells. Furthermore, the dopamine D2-like receptor agonist inhibited osteoclast formation induced by LPS *ex vivo* ^34^. This study indicates upregulation of D2R may inhibit *NFATc1* and *c-Fos* gene expression to suppress osteoclastogenesis (Fig. 7c). Further studies are needed to identify whether Dopa film directly activates D2R to promote osseointegration in a similar manner to the Dopa monomer, and examine whether other molecules are involved this process. Our findings also suggest the absence of a synergistic interaction between Dopa and ZA. It is possible that simple Dopa coating inhibits osteoclast differentiation-related genes by the same mechanism as ZA, which has been shown to inhibit osteoclast differentiation via suppression of *NFATc1* and *c-Fos* ^35,36^. In view of this finding, Dopa coating could be used in combination with osteogenic molecules–instead of inhibitors of bone resorption–to achieve a synergistic effect and obtain the desired extent of osseointegration.

## Conclusions

Simple Dopa coating promotes osseointegration of implants in osteoporotic rats in a similar manner to ZA modification. Dopa may promote osseointegration by inhibiting the expression of genes related to osteoclastogenesis. Surface modification with Dopa may provide a simple, promising strategy to improve new bone formation and remodelling around endosseous implants in the context of osteoporosis.

## Methods

### Titanium implant preparation and surface modifications

Commercial non-threaded titanium implants (2 mm diameter, 4 mm long) were sandblasted with 0.25–0.50 mm Al_2_O_3_ grit and acid etched (sandblasted and acid etched; SLA), followed by thermal etching with HCl/H_2_SO_4_ (Wego Jericom Biomaterials Co., Weihai, China). Samples were sterilized before use. For Dopa coating, implants were immersed in 5 mg/mL Dopa solution (Sigma-Aldrich, St Louis, MO, USA) in 10 mM Tris-HCl (pH 8.5) at 37 °C for 24 h. For combined Dopa and ZA modification, Dopa-coated implants were immersed in solution containing ZA (1 mg/mL; Sigma-Aldrich, St Louis, MO, USA) for 24 h. For simple ZA modification, implants without Dopa coating were immersed in solution containing ZA (1 mg/mL) for 24 h. Solutions were sterilized before use by filtration. Surface modified implants were rinsed with deionized water before use.

### Characterization of Dopa-coated titanium discs

Titanium implants were dried immediately before analysis; three independent measurements were made on each sample. Surface morphology was examined by scanning electron microscopy (SEM; S-4800, Hitachi, Ibaraki, Japan) at 15.0 kV. Surface elemental components were analyzed by X-ray photoelectron spectroscopy (XPS; ESCALAB 250Xi, Thermo Fisher Scientific, East Grinstead, UK) using a monochromatic Al K_α_ radiation source (1486.6 eV). Binding energy scales were calibrated with respect to C1s at 284.6 eV.

### Animal model of osteoporosis and implant surgery

Female 12-week-old Sprague-Dawley rats were housed under a 12 h:12 h light: dark cycle with free access to water and food. All animal procedures were approved by the ethics committee of Peking University Health Center, Beijing, China (license number: LA2015038) and the experiments were performed in accordance with the approved guidelines and regulations. Two rats were euthanized at 12 weeks after ovariectomy (OVX)^37^, and the femurs were harvested to confirm osteoporosis and compared with the femurs from sham operated rats by micro-computed tomography (micro-CT). The remaining rats were anaesthetized and implanted with a single titanium screw implant in the distal metaphysis of each femur as follows (*n* = 5 per group): SLA, SLA coated with Dopa (SLA-Dopa), SLA coated with ZA (SLA-ZA), and SLA coated with Dopa and ZA (SLA-Dopa + ZA). After 8 weeks, rats were euthanized and the femurs were harvested for further tests (Fig. 1e).

### Removal torque measurements

Peak resistance values to reverse torque rotation when loosening the implants from the distal femur specimens were recorded using a digital torque gauge (Mark-10, Copiague, New York, USA)^38^. In brief, the digital torque gauge was connected to the implant, and the same rotation rate of reverse torque was applied until loosening the fixation of the bone-implant interface occurred. The peak reverse-torque values required for complete loosening of the implants were recorded.

### Micro-CT analysis

Bone-implant surfaces and new bone formation were analyzed by micro-CT (Siemens, Munich, Germany; 80 kV, 500 μA, exposure time of 1500 ms); images were reconstructed using Cobra EXXIM software (EXXIM Computing Corp., Livermore, CA, USA). Quantitative analysis of the new bone formation around implants was carried out using Inveon Research Work-place (Siemens, Munich, Germany). The following micro-architecture parameters were assessed in the volume of interest area (2 mm around the implants): bone volume to total volume ratio (BV/TV), trabecular number (Tb.N), trabecular spacing (Tb.Sp) and trabecular thickness (Tb.Th). Three-dimensional images of the trabecular bone structures around the implants were generated using Inveon Research Work-place (Siemens) software.

### Histomorphometry

After micro-CT scanning, the femurs were dehydrated in an ascending ethanol series, and then processed for ground sectioning along the implant axis using Exakt Cutting and Grinding equipment (Exact Apparatebau, Norderstedt, Germany)^39^. After Levai-Laczko staining, the percentage of bone to implant contact (BIC) on the lateral side was calculated using a Bioquant Osteo Bone Biology Research System (BIOQUANT Image Analysis Corporation, Nashville, TN, USA); BIC was calculated as the length ratio of bone surface in direct contact with intra-bony implant surface^40^.

### Microarray analysis

Total RNA was extracted from the peri-implant bone attached to the implant surface and the surrounding bone using TRIzol reagent (Invitrogen Life Technologies, Carlsbad, CA, USA) according to the manufacturer’s protocol. Total RNA was hybridized to Affymetrix Rat Transcriptome 1.0 arrays (Affymetrix; Santa Clara, CA, USA) and subjected to microarray analysis (*n* = 3) by Shanghai Biotechnology Corporation.

### Statistical analysis

All data are mean ± standard deviation. *T-*tests or one-way analysis of variance (ANOVA) followed by the LSD *post hoc* test were used if the data followed a normal distribution. The rank sum test was used for non-normally distributed data. SPSS 17.0 software (SPSS Inc., Chicago, IL, USA) was used for analysis. *P-*values < 0.05 were considered statistically significant.

## Electronic supplementary material

Dataset 1
