# Supplementary material for: Simple 3,4-Dihydroxy-L-Phenylalanine Surface Modification Enhances Titanium Implant Osseointegration in Ovariectomized Rats
Source: Sci Rep. 2017 Dec 19;7:17849. doi: 10.1038/s41598-017-18173-5 (PMC5736607; doi:10.1038/s41598-017-18173-5)
Supplement: Supplementary file 1 — Dataset 1 [file 41598_2017_18173_MOESM1_ESM.doc]

Simple 3, 4-Dihydroxy-L-Phenylalanine Surface Modification Enhances Titanium Implant Osseointegration in Ovariectomized Rats

Ting Ma^1, +^, Xi-Yuan Ge^2, +^, Ke-Yi Hao^1^, Bi-Ru Zhang^1^, Xi Jiang^1^, Ye Lin^1,*^, Yu Zhang^1,*^

^1^ Department of Oral Implantology, Peking University School and Hospital of Stomatology, Beijing 100081, People’s Republic of China

^2^ Central Laboratory, Peking University School and Hospital of Stomatology, Beijing 100081, People’s Republic of China

Supplementary Data

**Supplementary Figure and Figure Legends**

**
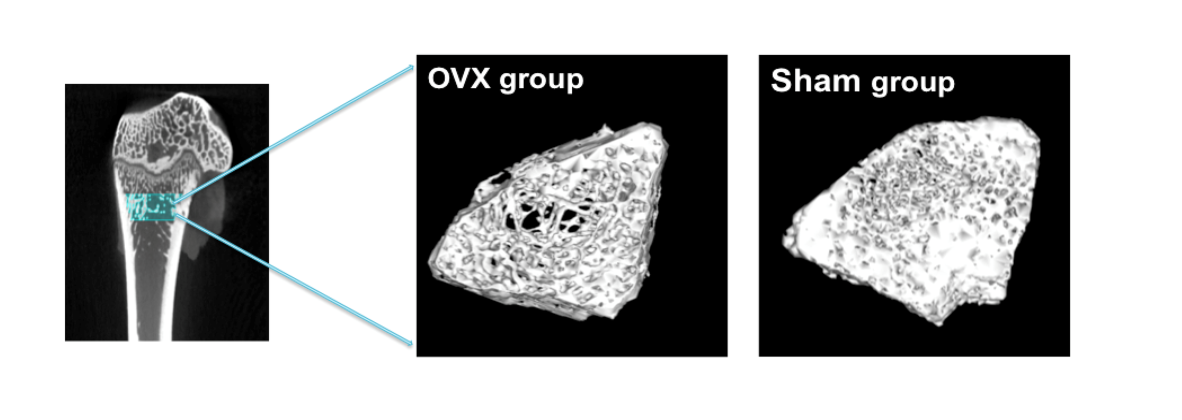
 Supplementary Figure 1.** Micro-CT micrographs of the femurs of rats at 12 weeks after OVX or sham surgery; OVX rats had expanded marrow cavities, disorganized trabecular architecture and less trabecular bone.
